# Modeling the Dynamics of Drug Spreading in China

**DOI:** 10.3390/ijerph18010288

**Published:** 2021-01-02

**Authors:** Haoxiang Tang, Mingtao Li, Xiangyu Yan, Zuhong Lu, Zhongwei Jia

**Affiliations:** 1 Department of Biomedical Engineering, College of Engineering, Peking University, Beijing 100871, China; thx1993@pku.edu.cn; 2 School of Mathematics, Taiyuan University of Technology, Taiyuan 030024, China; limingtao@tyut.edu.cn; 3 School of Public Health, Peking University, Beijing 100191, China; yanxiangyu@bjmu.edu.cn; 4 State Key Lab of Bioelectronics, School of Biological Science and Medical Engineering, Southeast University, Nanjing 210096, China; zhlu@seu.edu.cn; 5 Center for Intelligent Public Health, Institute for Artificial Intelligence, Peking University, Beijing 100191, China

**Keywords:** drug epidemic model, basic reproduction number, stability, sensitivity, China, numerical simulation

## Abstract

Drug abuse remains one of the major public health issues at the global level. In this article, we propose a drug epidemic model with a complete addiction–rehabilitation–recovery process, which allows the initiation of new users under the influence of drug addicts undergoing treatment and hidden drug addicts. We first conduct qualitative analyses of the dynamical behaviors of the model, including the existence and positivity of the solutions, the basic reproduction number, global asymptotic stabilities of both the drug-free and the drug-persistent equilibria, as well as sensitivity analysis. Then we use the model to predict the drug epidemic in China during 2020–2030. Finally, we numerically simulate the potential impact of intervention strategies on different drug users. The results show that the drug epidemic will decrease significantly during 2020−2030, and the most effective intervention strategy to eliminate drug epidemics is to strengthen the investigation and rehabilitation admission of hidden drug users.

## 1. Introduction

The phenomenon of drug abuse, which involves the consumption of illicit drugs and nonmedical use of prescription drugs, has become one of the global health issues threatening the safety and sustainability of human society in the 21st century. According to 2019 World Drug Report released by the United Nations Office on Drugs and Crime (UNODC), approximately 271 million people, which constituted 5.5% of the global population aged 15−64, had used drugs in 2016 [1]. From a historical perspective, the world has witnessed a 30% increase in the drug-using population ever since 2009 [1,2]. In terms of drug type, opioids remained the most lethal group, which resulted in around 66% of overdose-related deaths worldwide in 2017 [1]. The level of manufacture and trafficking of conventional drugs such as cocaine and cannabis remained high, and that of synthetic drugs such as methamphetamine and 3,4-methylenedioxy-n-methylamphetamine (MDMA) even soared in recent years. Some 35 million people suffered from drug use disorders and required treatment service around the world, and the death toll attributed to drug use totaled 585,000 in 2017 [1].

Despite its amelioration for two consecutive years, the drug situation in China remains a serious issue. According to the 2019 Report of Drug Situation in China, by the end of 2019, the number of drug users in China totaled 2.14 million (excluding the dead, those who went abroad, or those who remained abstinent for at least 3 years), which accounted for 0.16% of the total population [3]. In the same year, law enforcement agencies of China settled 83,000 drug-related crimes and seized 65.1 tons of drugs. Globally, around 43% of the People Who Inject Drugs (PWIDs) reside in three countries: China, Russia, and the United States [1]. All the evidence above aroused the need for global attention to the problem of drug abuse as well as targeted intervention to address this issue.

The mathematical model has long been an effective tool in the area of public health, and extensive research has been conducted in terms of drug users and drug-using behaviors. In the 1970s, the analogy between heroin use and communicable disease was proposed, which verified the validity and utility of the epidemiologic approach to studying heroin use [4,5,6]. This paved the way for the utilization of compartmental dynamic models, which could date back to 1926, when Kermack and McKendrick formulated the famous Susceptible–Infected–Recovered (SIR) compartmental model while studying the Great Plague of London during 1665−1666 and the 1906 plague in Bombay [7,8]. Compartmental dynamic modeling has undergone extensive development in the past decades and has proved itself as an effective tool in the research of infectious diseases, including influenza, smallpox, coronavirus, HIV, etc. [9,10,11,12,13]. In recent years, the application of compartmental dynamic models has been extended to other research fields, for instance, dynamics of the spread of alcoholism, cigarette smoking, internet virus, rumors, or drug-using behavior [14,15,16,17].

After several explorative research studies since the 1980s, a classic three-compartment dynamical model was proposed by White and Comiskey in 2006, which paved the way for drug epidemic models to come [18]. The authors utilized ordinary differential equations (ODE) systems and made calculations on several key aspects of the model, including basic reproduction number (R_0_), drug-free equilibrium, and drug-persistent equilibrium. Later on, with the aid of development in theories of nonlinear dynamic systems and computer-assisted simulation tools, plenty of research studies have sprung up worldwide, adding to this field of the drug epidemic model. A secondary analysis of the White-Comiskey model was conducted by Mulone et al. in 2009, who loosened the assumption of constant inflow rate [19]. Other modification studies, which were mostly heroin epidemic models, took advantage of various mathematical tools to account for practical factors. For example, delayed differential equations were used to simulate processes with known durations [20,21,22,23], partial differential equations were utilized to incorporate the effect of age or treatment duration [24,25,26,27,28,29], multi-layered models were proposed when population heterogeneity was involved [30,31,32,33], and stochastic differential equations were formulated to reflect unexpected fluctuations in reality [34,35,36,37,38]. In addition to theoretical analyses, some synthetic drug epidemic models were applied to real settings and fitted to historical data, most of which were based on methamphetamine epidemics in South Africa [39,40,41,42,43,44].

Despite these modeling efforts, few of them have investigated the drug situation in China [45]. Hence, it is our objective to model the scale and trend of the drug epidemic in China and thoroughly discuss specified intervention strategies. In this article, we formulate a drug epidemic model with a complete addiction–rehabilitation–recovery process. Unlike many other studies, we do not allow self-abstinence without treatment or any other unrealistic assumptions incompatible with the social background in China [46,47,48,49,50,51,52,53,54,55,56,57]. After qualitative analyses of the theoretical behaviors of the model system, we fit the model to historical data of drug abuse in China and make projections of the future. Efficiencies of various interventions are discussed. Our study is innovative from two aspects. First, a new version of the drug epidemic model is applied to the drug abuse epidemic in China. Second, new insights into intervention strategies to curtail the drug epidemic are provided.

This article is arranged as follows: In Section 2, we formulate the model and establish its basic properties. The existence and stability of the model equilibria was discussed in Section 3. In Section 4, sensitivity analysis and some numerical results were provided. Section 5 includes parameter estimation, model fitting, and projections, as well as simulations of intervention efficiencies. In Section 6, we conclude this paper with detailed discussions.

## 2. Model Assumptions, Formulations, and Basic Properties

### 2.1. Basic Assumptions

To study the dynamics of drug abuse in China, we formulate this drug epidemic model, in which the total population is divided into five mutually exclusive compartments: the susceptible population (S), light drug users (I_1_), drug addicts undergoing treatment (I_2_), hidden drug addicts (I_3_), and recovered individuals (R).

The population of interest is civilians aged between 15 and 64 years, while those outside of this age bracket are supposedly either too young to get in touch with drugs or old enough to mature out [18,58]. The susceptible compartment, which is defined as civilians with no history of drug use, receives a constant population inflow at an annual rate λ. This inflow takes into account young people reaching 15 years old as well as immigrants, and it is the only approach of population replenishment from outside the system. Light drug users (I_1_) are defined as individuals who tried drugs but have not reached addiction level, the criteria of which could be referred to Methods for the Identification of Drug Addiction issued by the Ministry of Public Security of China [59]. Drug addicts undergoing treatment (I_2_) are defined as drug users who have reached addiction level and are currently receiving detoxification rehabilitation. The drug rehabilitation system in China comprises compulsory-isolated detoxification centers, voluntary detoxification facilities, community detoxification and methadone maintenance treatment clinics (MMTs) [60,61,62,63], among which compulsory-isolated detoxification centers adopt in-patient bases and require isolation for two years, and other forms of detoxification facilities allow certain degrees of free movement. As a consequence, the I_2_ compartment is actually a mixture of patients receiving various forms of detoxification rehabilitation. Hidden drug addicts (I_3_) are defined as drug users who have reached addiction level and remained hidden to the law enforcement system, and the recovered compartment (R) is defined as individuals who have reached abstinence through detoxification treatment.

We assume homogeneous mixing and that the spread of drug-using behavior in the public can be modeled similar to an infectious disease. Susceptible individuals may initiate drug-using behavior and be converted to light drug users following interactions with drug addicts undergoing treatment (I_2_) or hidden drug addicts (I_3_), in which process we adopt a bilinear incidence rate. The compartment of drug addicts undergoing treatment (I_2_), which is a mixture of patients receiving various forms of rehabilitation, when regarded as a whole, manifests a lower mobility and an effective contact rate when compared with their hidden counterparts. Due to the illicit nature of drug-using behaviors in China, hidden drug addicts (I_3_) would try to evade law enforcement departments and are able to impose a larger impact on the transmission of drug addiction. We assume no fast progressor, which means a susceptible person must first become a light drug user before entering the compartments of addicts. While progressing to a drug addict, an individual would either be discovered and admitted to treatment facilities or remain hidden, corresponding to transfer rates $k_{1}$ and $k_{2}$, respectively. Hidden drug addicts (I_3_) could also be transferred to the I_2_ compartment at admission rate α, and those who finish rehabilitation will leave I_2_ and enter the recovered group R at recovery rate r. Without direct scientific proof of self-abstinence, we assume no other recovery approach without treatment in this model. Each compartment bears a natural death rate μ, and hidden drug addicts (I_3_) suffer from an additional death rate $\mu_{d}$ resulting from drug abuse [39,44]. The definitions of all compartment variables and parameters are listed in Table 1.

### 2.2. Model Formulations

**Figure 1:**
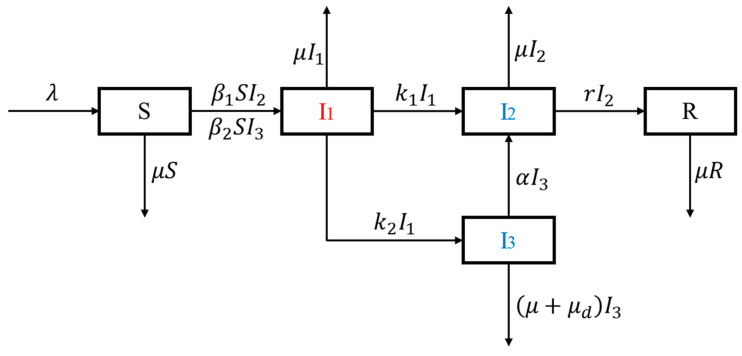
Flow diagram of the drug epidemic model.

The total population at time t is given by $N(t)=S(t)+I_{1}(t)+I_{2}(t)+I_{3}(t)+R(t)$. Based on the model assumptions above, we can obtain the model diagram (Figure 1) and the following set of nonlinear differential equations:

(1) $$\frac{dS}{dt}=\lambda -\beta_{1}SI_{2}-\beta_{2}SI_{3}-\mu S\;\frac{dI_{1}}{dt}=\beta_{1}SI_{2}+\beta_{2}SI_{3}-\left(k_{1}+k_{2}+\mu \right)I_{1}\;\frac{dI_{2}}{dt}=k_{1}I_{1}+\alpha I_{3}-\left(r+\mu \right)I_{2}\;\frac{dI_{3}}{dt}=k_{2}I_{1}-\left(\alpha +\mu +\mu_{d}\right)I_{3}\;\frac{dR}{dt}=rI_{2}-\mu R$$

with initial conditions $S\left(0\right)\geq 0,I_{1}\left(0\right)\geq 0,I_{2}\left(0\right)\geq 0,I_{3}\left(0\right)\geq 0,R\left(0\right)\geq 0$.

### 2.3. Existence and Uniqueness of the Solution

**Lemma 1**. *The model system admits a unique solution for non-negative initial conditions*$S\left(0\right)\geq 0,I_{1}\left(0\right)\geq 0,I_{2}\left(0\right)\geq 0,I_{3}\left(0\right)\geq 0,R\left(0\right)\geq 0$.

Proof Suppose $C\left(\left[a,b\right],{\mathbb{R}}^{5}\right)$ is the Banach space of continuous functions mapping the interval [a,b] into ${\mathbb{R}}^{5}$ with the topology of uniform convergence. System (1) with non-negative initial conditions can be considered as the following initial-value problem:

$$\left\{ \begin{array}{l} \dot{x}\left(t\right)=f\left(t,x\right)\\ x\left(t_{0}\right)=x_{0} \end{array}\right..$$

Let Ω be an open subset in ℝ×C and $f:\Omega \to {\mathbb{R}}^{5}$ is the mapping function of System (1). It is obvious that f(t,x) is continuous and Lipschitzian in x in each compact set in Ω. Since the initial point $\left(t_{0},x_{0}\right)\in \Omega$, according to Cauchy–Lipschitz Theorem, there is a unique solution of System (1) passing through $\left(t_{0},x_{0}\right)$ [64].

### 2.4. Feasible Region

The feasible region is an interval where System (1) will be analyzed, and it should be forward invariant for biological reasons. Thus, the following lemma will state the positive invariance and attractiveness of the system’s feasible region.

**Lemma 2**. *The feasible region of the model system is defined by*

(2) $$\Gamma =\left\{\left(S,I_{1},I_{2},I_{3},R\right)\in {\mathbb{R}}_{+}^{5};0\leq S+I_{1}+I_{2}+I_{3}+R\leq \frac{\lambda }{\mu }\right\}$$

*with initial conditions*$S\left(0\right)\geq 0,I_{1}\left(0\right)\geq 0,I_{2}\left(0\right)\geq 0,I_{3}\left(0\right)\geq 0,R\left(0\right)\geq 0$*,*Γ*is a positively invariant set, and it is attracting with regard to system (1) for all *t>0.

Proof Adding all the equations of System (1), we obtain

$$\frac{dN}{dt}=\lambda -\mu N-\mu_{d}I_{3}\leq \lambda -\mu N.$$

Solving this differential inequality, we have $0\leq N\left(t\right)\leq \frac{\lambda }{\mu }+\left[N\left(0\right)-\frac{\lambda }{\mu }\right]e^{-\mu t}$, where N(0) represents the sum of the initial values of all variables. Taking the limit as t→∞, we have that $0\leq N\leq \frac{\lambda }{\mu }$. Thus, the state variables will remain biologically meaningful in the feasible region Γ for all positive initial conditions, and Γ is positively invariant and attractive with respect to system (1). Hence, System (1) is well-posed mathematically and biologically in Γ, and it would suffice to study the dynamics of the system in Γ.

### 2.5. Positivity of Solutions

According to their biological meanings, all state variables and parameters are supposed to remain positive during the modeling period. In the following lemma, we will show that with positive initial conditions, each variable would remain non-negative for all t>0.

**Lemma 3**. *Given the initial conditions*$S\left(0\right)\geq 0,I_{1}\left(0\right)\geq 0,I_{2}\left(0\right)\geq 0,I_{3}\left(0\right)\geq 0,R\left(0\right)\geq 0$*, the solutions*$S\left(t\right),I_{1}\left(t\right),I_{2}\left(t\right),I_{3}\left(t\right)$*and*R(t)*will remain non-negative for all*t>0.

Proof Assume that $\bar{t}=\sup\left\{t>0:S>0,I_{1}>0,I_{2}>0,I_{3}>0,R>0\right\}\in \left[0,t\right]$. Thus, $\bar{t}>0$, and from the first equation of System (1), we have $\frac{dS}{dt}=\lambda -\beta_{1}SI_{2}-\beta_{2}SI_{3}-\mu S$.

Based on the Leibniz integral rule,

$$\frac{d}{dt}\int_{0}^{t}\left(\beta_{1}I_{2}+\beta_{2}I_{3}\right)ds=\beta_{1}I_{2}+\beta_{2}I_{3}+\int_{0}^{t}\frac{\partial }{\partial t}\left(\beta_{1}I_{2}+\beta_{2}I_{3}\right)ds\geq \beta_{1}I_{2}+\beta_{2}I_{3}\;\frac{d}{dt}\left[e^{\mu t+\int_{0}^{t}\left(\beta_{1}I_{2}+\beta_{2}I_{3}\right)ds}\right]\geq \left(\mu +\beta_{1}I_{2}+\beta_{2}I_{3}\right)e^{\mu t+\int_{0}^{t}\left(\beta_{1}I_{2}+\beta_{2}I_{3}\right)ds}\;\frac{d}{dt}\left[S\left(t\right)e^{\mu t+\int_{0}^{t}\left(\beta_{1}I_{2}+\beta_{2}I_{3}\right)ds}\right]\geq \left[\frac{dS\left(t\right)}{dt}+S\left(t\right)\cdot \left(\mu +\beta_{1}I_{2}+\beta_{2}I_{3}\right)\right]e^{\mu t+\int_{0}^{t}\left(\beta_{1}I_{2}+\beta_{2}I_{3}\right)ds}=\lambda e^{\mu t+\int_{0}^{t}\left(\beta_{1}I_{2}+\beta_{2}I_{3}\right)ds}\;S\left(\bar{t}\right)e^{\mu \bar{t}+\int_{0}^{\bar{t}}\left(\beta_{1}I_{2}+\beta_{2}I_{3}\right)ds}-S\left(0\right)\geq \int_{0}^{\bar{t}}\lambda e^{\mu \bar{t}+\int_{0}^{\bar{t}}\left(\beta_{1}I_{2}+\beta_{2}I_{3}\right)ds}d\bar{t},\;S\left(\bar{t}\right)\geq e^{-\left[\mu \bar{t}+\int_{0}^{\bar{t}}\left(\beta_{1}I_{2}+\beta_{2}I_{3}\right)ds\right]}\left[S\left(0\right)+\int_{0}^{\bar{t}}\lambda e^{\mu \bar{t}+\int_{0}^{\bar{t}}\left(\beta_{1}I_{2}+\beta_{2}I_{3}\right)ds}d\bar{t}\right]>0.$$

From the second equation of System (1), we obtain

$$\frac{dI_{1}}{dt}\geq -\left(k_{1}+k_{2}+\mu \right)I_{1}.$$

Solving the differential inequality, we have:

$$I_{1}\left(t\right)\geq I_{1}\left(0\right)e^{-\left(k_{1}+k_{2}+\mu \right)t}>0.$$

Similarly, it can be shown that $I_{2}\left(t\right)>0,I_{3}\left(t\right)>0$ and R(t)>0 for all t>0.

## 3. Model Equilibria Analysis

### 3.1. The Basic Reproduction Number

In this model, the basic reproduction number is defined as the number of secondary drug users converted from susceptible individuals by a single drug addict (either hidden or undergoing treatment) introduced into a totally susceptible population during his entire addiction period [8,54]. The basic reproduction number plays a vital role in determination of the existence of the drug epidemic, as well as the analysis of dynamics of the model. In this section, we will obtain the formula of the basic reproduction number with the next-generation matrix method [65].

First, we rearrange the equations of System (1) according to the order of $I_{1},I_{2},I_{3},S,R$ and let $x=\left(I_{1},I_{2},I_{3},S,R\right)$ be the solution to the system; then, System (1) can be rewritten as:

$$\frac{dx}{dt}=ℱ-V=\left[\begin{array}{c} \beta_{1}SI_{2}+\beta_{2}SI_{3}\\ 0\\ 0\\ 0\\ 0 \end{array}\right]-\left[\begin{array}{c} \left(k_{1}+k_{2}+\mu \right)I_{1}\\ \left(r+\mu \right)I_{2}-k_{1}I_{1}-\alpha I_{3}\\ \left(\alpha +\mu +\mu_{d}\right)I_{3}-k_{2}I_{1}\\ \beta_{1}SI_{2}+\beta_{2}SI_{3}+\mu S-\lambda \\ \mu R-rI_{2} \end{array}\right]$$

where ℱ represents the new initiate terms, and V corresponds to the interior transfer terms. The corresponding linearized matrices of ℱ and V evaluated at the drug-free equilibrium $E_{0}=\left(\frac{\lambda }{\mu },0,0,0,0\right)$ are

$$F=\left[\begin{array}{ccc} 0 & \beta_{1}S_{0} & \beta_{2}S_{0}\\ 0 & 0 & 0\\ 0 & 0 & 0 \end{array}\right],\;V=\left[\begin{array}{ccc} k_{1}+k_{2}+\mu & 0 & 0\\ -k_{1} & r+\mu & -\alpha \\ -k_{2} & 0 & \alpha +\mu +\mu_{d} \end{array}\right].$$

The next-generation matrix is given by $FV^{-1}=\left[\begin{array}{ccc} A_{11} & A_{12} & A_{13}\\ 0 & 0 & 0\\ 0 & 0 & 0 \end{array}\right]$, where

$$A_{11}=\left[\frac{\left(\alpha +\mu +\mu_{d}\right)k_{1}+\alpha k_{2}}{\left(k_{1}+k_{2}+\mu \right)\left(r+\mu \right)\left(\alpha +\mu +\mu_{d}\right)}\beta_{1}+\frac{k_{2}}{\left(k_{1}+k_{2}+\mu \right)\left(\alpha +\mu +\mu_{d}\right)}\beta_{2}\right]\frac{\lambda }{\mu },$$

(3) $$A_{12}=\frac{\beta_{1}\lambda }{\mu \left(r+\mu \right)},A_{13}=\left[\frac{\alpha \beta_{1}}{\left(r+\mu \right)\left(\alpha +\mu +\mu_{d}\right)}+\frac{\beta_{2}}{\alpha +\mu +\mu_{d}}\right]\frac{\lambda }{\mu }.$$

Thus, the basic reproduction number $R_{0}$ is defined as the spectral radius of the next-generation matrix, which is the largest absolute value of its eigenvalues:

$$R_{0}=\rho \left(FV^{-1}\right)=\left[\frac{\left(\alpha +\mu +\mu_{d}\right)k_{1}+\alpha k_{2}}{\left(k_{1}+k_{2}+\mu \right)\left(r+\mu \right)\left(\alpha +\mu +\mu_{d}\right)}\beta_{1}+\frac{k_{2}}{\left(k_{1}+k_{2}+\mu \right)\left(\alpha +\mu +\mu_{d}\right)}\beta_{2}\right]\frac{\lambda }{\mu }.$$

The epidemiological meaning of this formula can be understood in the following way. The first term of the expression above represents the role of drug addicts undergoing treatment, i.e., when a drug addict in treatment is introduced into a wholly susceptible population with a size of $\frac{\lambda }{\mu }$, the number of light drug users converted from susceptible individuals under his/her influence in unit time is $\frac{\beta_{1}\lambda }{\mu }$, the proportion of which who progress to $I_{2}$ compartment is $\frac{k_{1}}{k_{1}+k_{2}+\mu }$, whose average addiction period is $\frac{1}{r+\mu }$; hence, the number of $I_{2}$ directly generated during this period is $\frac{k_{1}\beta_{1}\lambda }{\mu \left(k_{1}+k_{2}+\mu \right)\left(r+\mu \right)}$. On the other hand, the proportion of $I_{1}$ who progress to $I_{3}$ is $\frac{k_{2}}{k_{1}+k_{2}+\mu }$, and the proportion of $I_{3}$ who progress to $I_{2}$ is $\frac{\alpha }{\alpha +\mu +\mu_{d}}$; hence, the number of $I_{2}$ generated through $I_{3}$ during this period is obtained as $\frac{\alpha k_{2}\beta_{1}\lambda }{\mu \left(k_{1}+k_{2}+\mu \right)\left(r+\mu \right)\left(\alpha +\mu +\mu_{d}\right)}$. Adding these two parts gives the first term of the formula. The latter term can be explained in a similar way, which represents the role of hidden drug addicts. When a hidden drug addict is introduced into this wholly susceptible population, the number of light drug users converted from susceptible individuals under his/her influence in unit time is $\frac{\beta_{2}\lambda }{\mu }$, the proportion of which who progress to $I_{2}$ compartment is $\frac{k_{2}}{k_{1}+k_{2}+\mu }$, and the average addiction period of $I_{3}$ is $\frac{1}{\alpha +\mu +\mu_{d}}$; hence, the number of $I_{2}$ generated during this period is obtained as $\frac{k_{2}\beta_{2}\lambda }{\mu \left(k_{1}+k_{2}+\mu \right)\left(\alpha +\mu +\mu_{d}\right)}$. To sum up, the formula of basic reproduction number represents the overlay of the influence of drug addicts undergoing treatment and hidden drug addicts.

### 3.2. Existence of the Equilibria

In order to investigate the existence of the drug-free equilibrium and the drug-persistent equilibrium, we set the left side of the equations of System (1) at zero:

(4) $$\lambda -\beta_{1}SI_{2}-\beta_{2}SI_{3}-\mu S=0\;\beta_{1}SI_{2}+\beta_{2}SI_{3}-\left(k_{1}+k_{2}+\mu \right)I_{1}=0\;k_{1}I_{1}+\alpha I_{3}-\left(r+\mu \right)I_{2}=0\;k_{2}I_{1}-\left(\alpha +\mu +\mu_{d}\right)I_{3}=0\;rI_{2}-\mu R=0.$$

Through direct calculations, we obtain:

$$\frac{\left[\left(\alpha +\mu +\mu_{d}\right)k_{1}+\alpha k_{2}\right]\beta_{1}+\left(r+\mu \right)k_{2}\beta_{2}}{\left(r+\mu \right)\left(\alpha +\mu +\mu_{d}\right)}SI_{1}=\left(k_{1}+k_{2}+\mu \right)I_{1}.$$

1. When $I_{1}=0$, it is easy to acquire $I_{2}=I_{3}=R=0$, $S=\frac{\lambda }{\mu }$. At this point, $N=S=\frac{\lambda }{\mu }$, and $\frac{dN}{dt}=\lambda -\mu N-\mu_{d}I_{3}=0$ is also satisfied. We refer to this point as the drug-free equilibrium $E_{0}=\left(\frac{\lambda }{\mu },0,0,0,0\right)$.
2. When $I_{1}\neq 0$, it is obvious that $S^{*}=\frac{\left(r+\mu \right)\left(\alpha +\mu +\mu_{d}\right)\left(k_{1}+k_{2}+\mu \right)}{\left[\left(\alpha +\mu +\mu_{d}\right)k_{1}+\alpha k_{2}\right]\beta_{1}+\left(r+\mu \right)k_{2}\beta_{2}}$ and $I_{1}^{*}=\frac{\lambda -\mu S^{*}}{k_{1}+k_{2}+\mu },I_{2}^{*}=\frac{\left(\alpha +\mu +\mu_{d}\right)k_{1}+\alpha k_{2}}{\left(r+\mu \right)\left(\alpha +\mu +\mu_{d}\right)}I_{1}^{*}$,$I_{3}^{*}=\frac{k_{2}}{\alpha +\mu +\mu_{d}}I_{1}^{*},R^{*}=\frac{r}{\mu }I_{2}^{*}$. At this point, we obtain $N=S^{*}+I_{1}^{*}+I_{2}^{*}+I_{3}^{*}+R^{*}=\frac{\left[\left(\alpha +\mu +\mu_{d}\right)\left(k_{1}+\mu \right)+k_{2}\left(\alpha +\mu \right)\right]\lambda +k_{2}\mu \mu_{d}S^{*}}{\mu \left(k_{1}+k_{2}+\mu \right)\left(\alpha +\mu +\mu_{d}\right)}$, and it can be easily verified that $\frac{dN}{dt}=\lambda -\mu N-\mu_{d}I_{3}=0$ also holds. We refer to this point as the unique drug-persistent equilibrium $E^{*}=\left(S^{*},I_{1}^{*},I_{2}^{*},I_{3}^{*},R^{*}\right)$. For biological reasons, it requires that $S^{*}<\frac{\lambda }{\mu }$, which corresponds to the condition that $R_{0}>1$.

### 3.3. Global Stability of the Drug-Free Equilibrium

**Theorem 1**. *The drug-free equilibrium*$E_{0}$*is globally asymptotically stable when *$R_{0}\leq 1$.

Proof See Appendix A for a detailed proof of Theorem 1. 

### 3.4. Global Stability of the Drug-Persistent Equilibrium

**Theorem 2**. *The drug-persistent equilibrium*$E^{*}$is globally asymptotically stable when$R_{0}>1$.

Proof See Appendix B for a detailed proof of Theorem 2.

## 4. Sensitivity and Numerical Simulations

### 4.1. Sensitivity Analysis

Due to the critical role of the basic reproduction number $R_{0}$ in determination of the persistence of the drug epidemic, it is of vital importance to identify the most effective approach to bring $R_{0}$ down to below one. In this section, we calculate the normalized forward sensitivity index (NFSI) of $R_{0}$ with respect to each parameter following Arriola and Hyman [66]. Considering their biological and epidemiological meanings, the changes of some parameters are either impractical or unethical (demographic parameters or inherent progression rates, etc.). Hence, we have identified four parameters of interest and calculated their NFSIs:

$$A_{\beta_{1}}=\left|\frac{\partial R_{0}}{\partial \beta_{1}}\right|\cdot \left|\frac{\beta_{1}}{R_{0}}\right|=\frac{\left[\left(\alpha +\mu +\mu_{d}\right)k_{1}+\alpha k_{2}\right]\beta_{1}}{\left[\left(\alpha +\mu +\mu_{d}\right)k_{1}+\alpha k_{2}\right]\beta_{1}+k_{2}\left(r+\mu \right)\beta_{2}},\;A_{\beta_{2}}=\left|\frac{\partial R_{0}}{\partial \beta_{2}}\right|\cdot \left|\frac{\beta_{2}}{R_{0}}\right|=\frac{k_{2}\left(r+\mu \right)\beta_{2}}{\left[\left(\alpha +\mu +\mu_{d}\right)k_{1}+\alpha k_{2}\right]\beta_{1}+k_{2}\left(r+\mu \right)\beta_{2}},\;A_{k_{1}}=\left|\frac{\partial R_{0}}{\partial k_{1}}\right|\cdot \left|\frac{k_{1}}{R_{0}}\right|=\frac{k_{1}}{k_{1}+k_{2}+\mu }\cdot \frac{\left|\left(\alpha +\mu +\mu_{d}\right)\left(k_{1}+\mu \right)\beta_{1}-k_{2}\left[\alpha \beta_{1}+\left(r+\mu \right)\beta_{2}\right]\right|}{\left[\left(\alpha +\mu +\mu_{d}\right)k_{1}+\alpha k_{2}\right]\beta_{1}+k_{2}\left(r+\mu \right)\beta_{2}},\;A_{\alpha }=\left|\frac{\partial R_{0}}{\partial \alpha }\right|\cdot \left|\frac{\alpha }{R_{0}}\right|=\frac{\alpha }{\alpha +\mu +\mu_{d}}\cdot \frac{\left|\left(\mu +\mu_{d}\right)k_{2}\beta_{1}-\left(r+\mu \right)k_{2}\beta_{2}\right|}{\left[\left(\alpha +\mu +\mu_{d}\right)k_{1}+\alpha k_{2}\right]\beta_{1}+k_{2}\left(r+\mu \right)\beta_{2}}.$$

The signs of the numerators of $A_{k_{1}}$ and $A_{\alpha }$ depend on the final values of the parameters, and it is easy to prove that all NFSIs are lower than one. According to their biological meanings, we have $\beta_{2}\gg \beta_{1}$ for most cases; hence, it seems obvious that $A_{\beta_{2}}>A_{\beta_{1}}$. That is to say, $R_{0}$ is more sensitive to changes in $\beta_{2}$ than those in $\beta_{1}$. The relative sizes of $A_{\beta_{2}}$, $A_{k_{1}}$, and $A_{\alpha }$ are yet to be determined, depending on the exact value of each parameter.

### 4.2. Simulation of Sensitivity

With the aim of illustrating the sensitivity of the basic reproduction number $R_{0}$ with respect to the parameters of interest, we conduct a series of numerical simulations based on artificial training parameters. The parameter ranges are chosen to accommodate reasonable biological meanings, and extra attention is paid when the basic reproduction number $R_{0}$ crosses one, which acts as the threshold for the persistence of the drug epidemic.

(1) Comparison between $\beta_{1}$ and $\beta_{2}$

**Figure 2:**
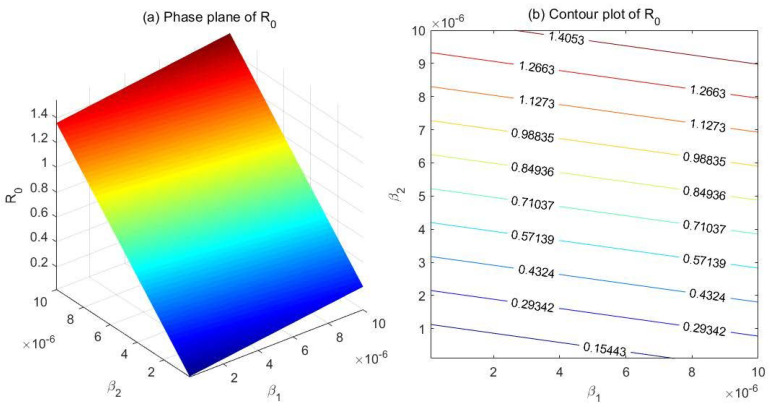
Phase plane (**a**) and contour plot (**b**) of the basic reproduction number $R_{0}$ with respect to $\beta_{1}$ (effective contact rate between drug addicts undergoing treatment and the susceptible) and $\beta_{2}$ (effective contact rate between hidden drug addicts and the susceptible).

Fix the parameters at λ=100, μ=0.007, $\mu_{d}=0.025$, $k_{1}=0.05$, $k_{2}=0.2$, α=0.05, and r=0.5. Set $\beta_{1}\in \left[1e-7,1e-5\right]$ and $\beta_{2}\in \left[1e-7,1e-5\right]$, and draw the phase plane as well as the contour plot of $R_{0}$ with respect to $\beta_{1}$ and $\beta_{2}$ (Figure 2). According to the phase plane (Figure 2a), the value of $R_{0}$ decreases sharply as $\beta_{2}$ decreases, but the change of $\beta_{1}$ has a significantly smaller impact on $R_{0}$. The contour plot (Figure 2b) demonstrates similar results, which indicates that $R_{0}$ is far more sensitive to $\beta_{2}$ than to $\beta_{1}$ in this case.

(2) Comparison between $\beta_{2}$ and $k_{1}$

**Figure 3:**
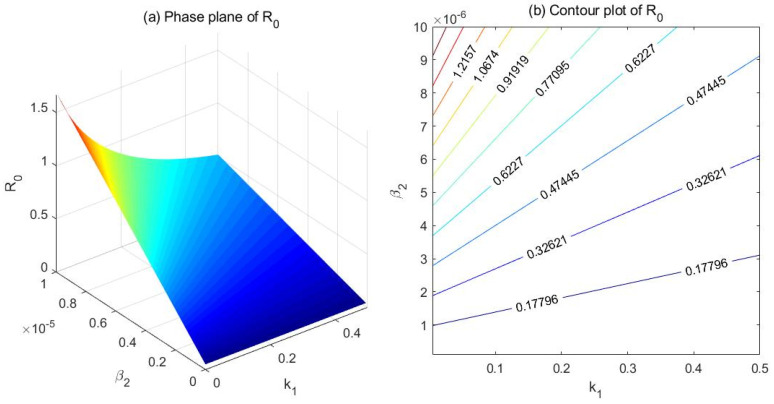
Phase plane (**a**) and contour plot (**b**) of the basic reproduction number $R_{0}$ with respect to $\beta_{2}$ (effective contact rate between hidden drug addicts and the susceptible) and $k_{1}$ (transfer rate from light drug users to drug addicts undergoing treatment).

Fix the parameters at λ=100, μ=0.007, $\mu_{d}=0.025$, $k_{2}=0.2$, α=0.05, r=0.5, and $\beta_{1}=1e-6$. Set $\beta_{2}\in \left[1e-7,1e-5\right]$ and $k_{1}\in \left[0.005,0.5\right]$, and draw the phase plane as well as the contour plot of $R_{0}$ with respect to $\beta_{2}$ and $k_{1}$ (Figure 3). Both the phase plane (Figure 3a) and the contour plot (Figure 3b) indicate that $\beta_{2}$ is more effective than $k_{1}$ in controlling $R_{0}$. In this case, increasing $k_{1}$ also lowers $R_{0}$ to some extent; note that $R_{0}$ is negatively correlated with $k_{1}$.

(3) Comparison between $\beta_{2}$ and α

**Figure 4:**
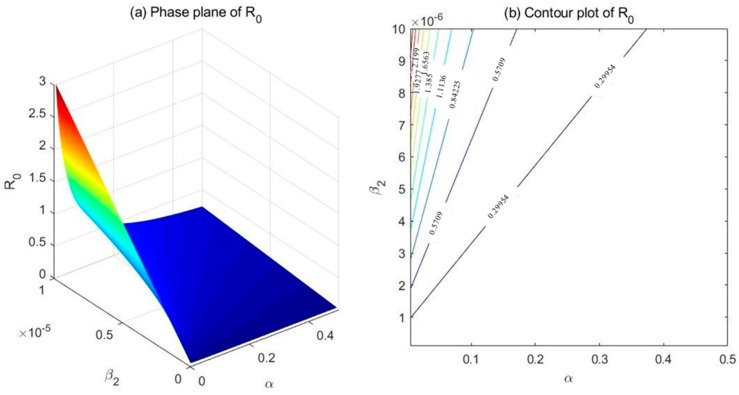
Phase plane (**a**) and contour plot (**b**) of the basic reproduction number $R_{0}$ with respect to $\beta_{2}$ (effective contact rate between hidden drug addicts and the susceptible) and α (transfer rate from hidden drug addicts to drug addicts undergoing treatment).

Fix the parameters at λ=100, μ=0.007, $\mu_{d}=0.025$, $k_{1}=0.05$, $k_{2}=0.2$, r=0.5, and $\beta_{1}=1e-6$. Set $\beta_{2}\in \left[1e-7,1e-5\right]$ and α∈[0.005,0.5], and draw the phase plane as well as the contour plot of $R_{0}$ with respect to $\beta_{2}$ and α (Figure 4). As demonstrated in the phase plane (Figure 4a) and the contour plot (Figure 4b), the relative effectiveness of $\beta_{2}$ and α in controlling $R_{0}$ seems debatable. In the top-left corner of the contour plot, which corresponds to lower values of α and higher values of $\beta_{2}$, α turns out more efficient in adjusting the basic reproduction number $R_{0}$.

### 4.3. Simulation of Model Equilibria

With the aid of numerical simulation tools, we are also able to illustrate the global stability of the model equilibria. For this purpose, we fix the parameters and obtain five distinct solutions for five different sets of initial values.

(1) Drug-free equilibrium

**Figure 5:**
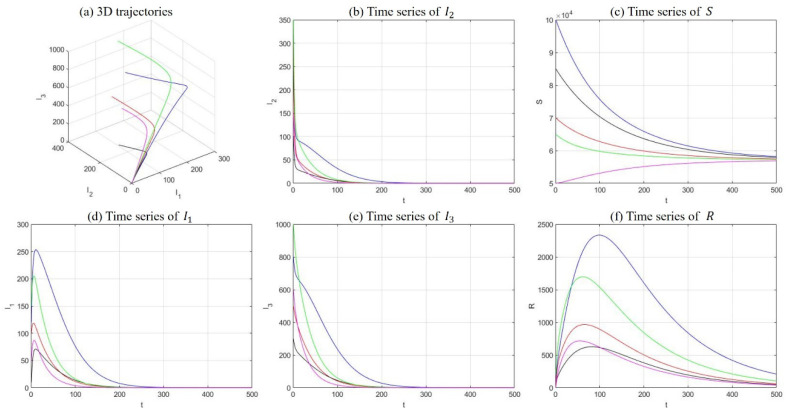
Numerical simulations with five sets of initial conditions with respect to a drug-free equilibrium, including 3D trajectories in the $I_{1}-I_{2}-I_{3}$ space (**a**) and long-term time-series plots of drug addicts undergoing treatment (**b**), susceptible individuals (**c**), light drug users (**d**), hidden drug addicts (**e**), and the recovered individuals (**f**).

Fix the parameters at λ=400, μ=0.007, $\mu_{d}=0.025$, $k_{1}=0.05$, $k_{2}=0.2$, r=0.5, α=0.05, $\beta_{1}=1e-7$, and $\beta_{2}=1e-6$. The solutions of five sets of initial values were demonstrated in Figure 5, including 3D trajectories in the $I_{1}-I_{2}-I_{3}$ space and long-term time-series plot of each compartment. In this case, $R_{0}=0.5498$ and the model equilibrium manifests as a drug-free equilibrium. The statement is supported by Figure 5, where the number of susceptible individuals stabilizes at somewhere above zero, and all other compartments fade away with time.

(2) Drug-persistent equilibrium

**Figure 6:**
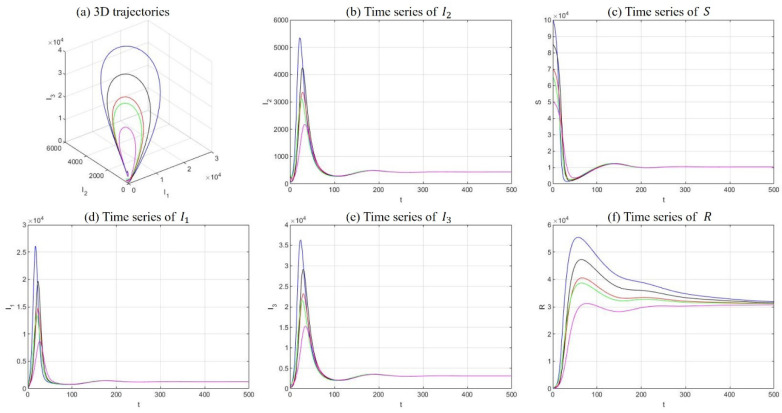
Numerical simulations with five sets of initial conditions with respect to a drug-persistent equilibrium, including 3D trajectories in the $I_{1}-I_{2}-I_{3}$ space (**a**) and long-term time-series plots of drug addicts undergoing treatment (**b**), susceptible individuals (**c**), light drug users (**d**), hidden drug addicts (**e**), and the recovered individuals (**f**).

Fix the parameters at λ=400, μ=0.007, $\mu_{d}=0.025$, $k_{1}=0.05$, $k_{2}=0.2$, r=0.5, α=0.05, $\beta_{1}=1e-6$, and $\beta_{2}=1e-5$. The solutions of five sets of initial values were demonstrated in Figure 6. In this case, $R_{0}=5.4985$ and the model equilibrium manifests as a drug-persistent equilibrium. This statement is supported by Figure 6, where all compartments persist and stabilize somewhere above zero.

## 5. Application of the Model

Based on the qualitative results of dynamical behaviors acquired above, we now apply the model to the drug epidemic in real world. Since our model possess a complete addiction–rehabilitation–recovery process, and its basic assumptions are more compatible with the current situation in mainland China, we fit our model to the historical data of drug users in China and aim to predict the scale and trend of drug users in the near future as well as analyze the potential effect of various intervention strategies.

### 5.1. Data Source and Variables

The most comprehensive and publicly available official sources are the Annual Report of Drug Situation in China and Annual Report on Drug Control in China released by the National Narcotics Control Committee (NNCC) [67]. Since 2015, the reports have changed their scopes of statistics and provided the numbers of all existing drug users (excluding those who had died, emigrated, or remained abstinent for three or more years) instead of the numbers of cumulative totals previously reported [3,68]. According to the Law of Drug Control and the Drug Rehabilitation Ordinance, all drug users identified will receive their corresponding type of rehabilitation treatment; thus, we can safely conclude that the numbers of all existing drug users released by the NNCC correspond to the compartment of drug addicts undergoing treatment ($I_{2}$) in our model. The numbers of former drug users who had remained abstinent for three or more years reported by the NNCC approximately correspond to the compartment of individuals who have reached abstinence through detoxification treatment (R). Without a direct data source, the initial value of hidden drug addicts ($I_{3}$) can be ascertained through the initial value of $I_{2}$ compartment and the explicit-to-implicit ratio of 1:4 reported by the NNCC and the existing literature [62,68,69,70]. Likewise, the initial value of light drug users is estimated based on dynamical relationships with neighboring compartments. Finally, the initial value of the susceptible individuals (S) is obtained through subtracting all other compartments from the total population aged 15−64 recorded by the National Bureau of Statistics [71]. The historical numbers of population aged 15−64, existing drug users, and former drug users who had remained abstinent for three or more years are listed in Table 2.

### 5.2. Parameter Estimation

We obtain demographic parameters mainly from official data released by the authorities, among which the natural death rate μ was acquired from the website of The National Bureau of Statistics, and inflow rate into the susceptible compartment λ was ascertained through the equation that *Net population growth = Inflow–Death* [71]. Other parameters with explicit sources include the additional death rate of hidden drug addicts $\mu_{d}$ and the progression rate from light drug users to hidden drug addicts $k_{2}$ [23,25,39,44,72]. Without direct data, the recovery rate was ascertained according to the rehabilitation durations of the detoxification facilities [60]. Other parameters involve implicit processes that are hard to study directly and will need to be ascertained through fitting procedures. The potential ranges of the parameters were chosen based on dynamical relationships between neighboring compartments and parameters with similar roles in other modeling studies as well [23,25,39,44,60,72]. For instance, with around 1 billion susceptible individuals and 10 million hidden drug addicts at the starting point, the incidence rate of new initiates under the influence of hidden drug addicts shall not exceed 1 million/year (which approximates the number of light drug users at the initial phase). According to the bilinear incidence rate adopted, we obtain that an effective contact rate of $\beta_{2}<1e-6$ /10 thousand people*year from this inequality. We list the parameter units, value ranges, final values used, and their sources in Table 3.

### 5.3. Model Fitting and Projections

**Figure 7:**
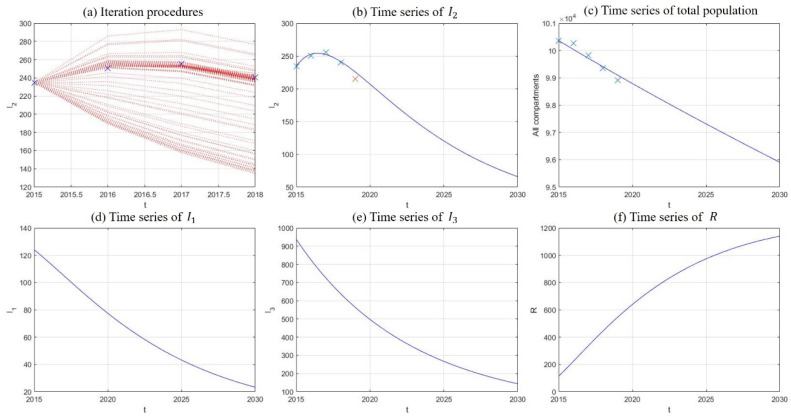
Model fitting and projection results, including iteration procedures of curve fitting (**a**) and time-series plots of drug addicts undergoing treatment (**b**), sum of all compartments (**c**), light drug users (**d**), hidden drug addicts (**e**), and recovered individuals (**f**).

In this section, we fit our model to historical data of drug abuse in China, where reported numbers of existing drug users during 2015–2019 were used to model the growth of drug addicts undergoing treatment. Instead of using all five data points in the curve-fitting procedure, we set the point of 2019 aside and used it for verification. Least square curve fitting was realized through the fminsearch function in the Matlab program (Mathworks Corp, Natick, MA, USA), and numerical solutions of the model system were obtained by the Runge–Kutta method of order 4. The iteration procedures of fitting are plotted in Figure 7a, where blue crosses are historical data of existing drug users during 2015–2018. From Figure 7b to Figure 7f, time series of $I_{2},N,I_{1},I_{3}$ and R are plotted along with their projections until 2030. Blue crosses in Figure 7b have the same meanings as in Figure 7a, and the red cross marks the corresponding data in 2019. Crosses in Figure 7c represent historical data of population aged 15−64 recorded by The National Bureau of Statistics, which acted as verification with the sum of all compartments. It can be seen from the results in Figure 7 that the numbers of light drug users, drug addicts undergoing treatment, and hidden drug addicts experience decreases of 81.22%, 71.98%, and 84.69% respectively in 15 years, so long as the model dynamics remain unperturbed. The sum of all compartments experiences a mild decrease of 4.44%, which is in consistent with the tendencies of historical data. The final values of the parameters acquired through curve fitting are listed in Table 3, and the corresponding basic reproduction number was calculated as $R_{0}=0.087256$, which is quite low and accords with the rapid decreases observed in the number of drug addicts undergoing treatment and hidden drug addicts.

### 5.4. Evaluation of Intervention Strategies

Despite the delightful tendency of decrease generated by model simulation in the previous section, it is still the objective of policy makers and public health workers to further shrink the scale of drug epidemic. In this section, we present hypothesized results of several potential intervention strategies through numerical simulation and compare their effects on drug control.

(1) Intervention 1: anti-drug education and propaganda

**Figure 8:**
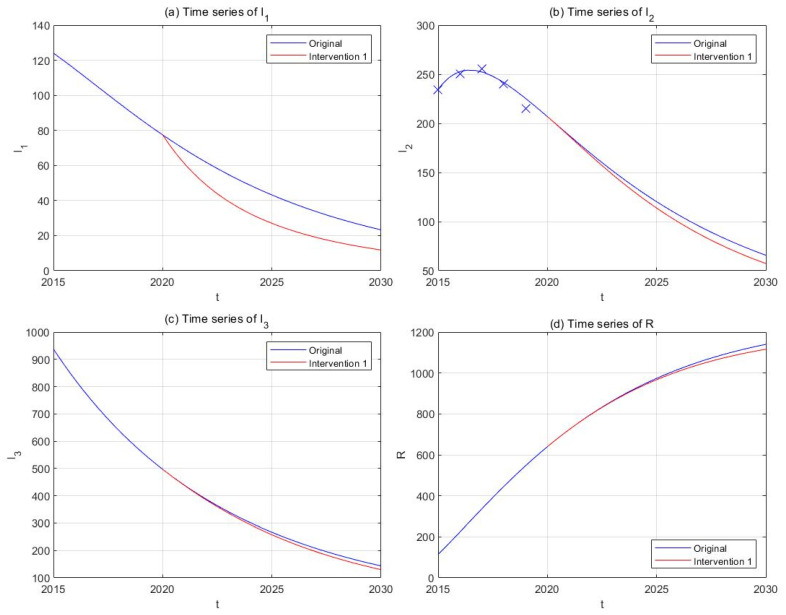
The effect of Intervention 1 (anti-drug education and propaganda, red curves) in comparison with the original drug situation (blue curves). Subplots include time-series plots of light drug users (**a**), drug addicts undergoing treatment (**b**), hidden drug addicts (**c**), and the recovered individuals (**d**).

Previous studies have shown that a misconception of drugs (86−90%) and peer influence (13−44%) are the most common reasons for initiating drug use among Chinese drug users [73,74]. School-based anti-drug education or preventive propaganda through media publicity has been implemented in China in the past decades and proved itself as an effective tool against drug epidemic [75]. Strengthening anti-drug propaganda could correct misunderstandings of drugs among the susceptible individuals and lower the risk of first exposure to illicit drugs. We assume that the effective contact rates $\beta_{1}$ and $\beta_{2}$ are inversely proportional to the intensity of anti-drug education and propaganda, and that doubling the frequency and budgets of such activities could lower $\beta_{1}$ and $\beta_{2}$ by 50%. Suppose this strategy starts to take effect since the end of 2020, and plot the new curves of the model solution in parallel with the original ones (Figure 8). The results show that the implementation of this intervention will be able to lower the number of light drug users (−49.27%) and drug addicts undergoing treatment (−12.79%) by 2030, but its effect on hidden addicts (−9.58%) is relatively smaller.

(2) Intervention 2: moderate anti-drug propaganda

**Figure 9:**
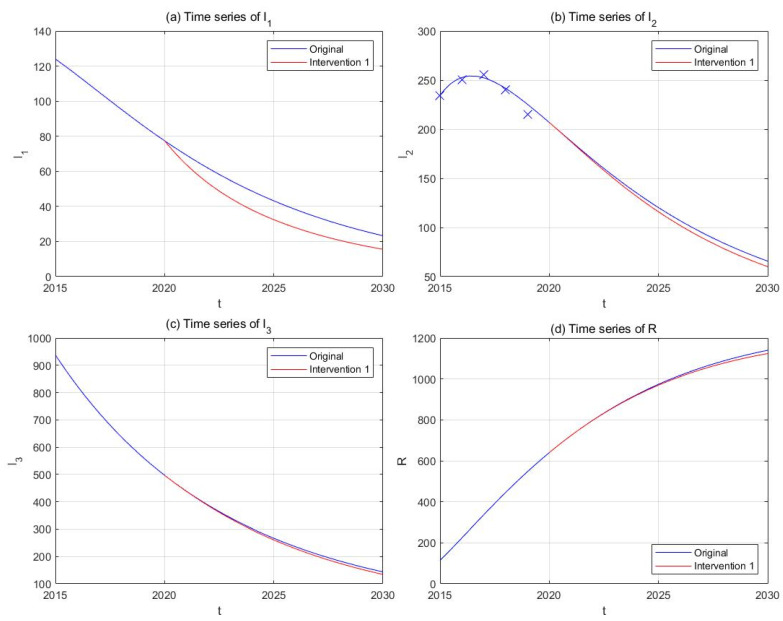
The effect of Intervention 2 (regional anti-drug propaganda, red curves) in comparison with the original drug situation (blue curves). Subplots include time series plots of light drug users (**a**), drug addicts undergoing treatment (**b**), hidden drug addicts (**c**), and the recovered individuals (**d**).

Accounting for the limited resources available for anti-drug education and propaganda, as well as the huge number of susceptible individuals, we might consider an alternative approach instead of an amplification of 100% in the intensities of such activities. Hence, in this intervention strategy, the frequency and budgets of anti-drug education and propaganda are increased by 50%, which correspond to a 33% decrease in effective contact rates $\beta_{1}$ and $\beta_{2}$. We repeat the rest of the procedures and obtain simulation results in Figure 9. The results showed that the number of light drug users will be lowered by 33.20% compared to the original curve, and the impact on hidden drug addicts is minimal.

(3) Intervention 3: investigation and admission

**Figure 10:**
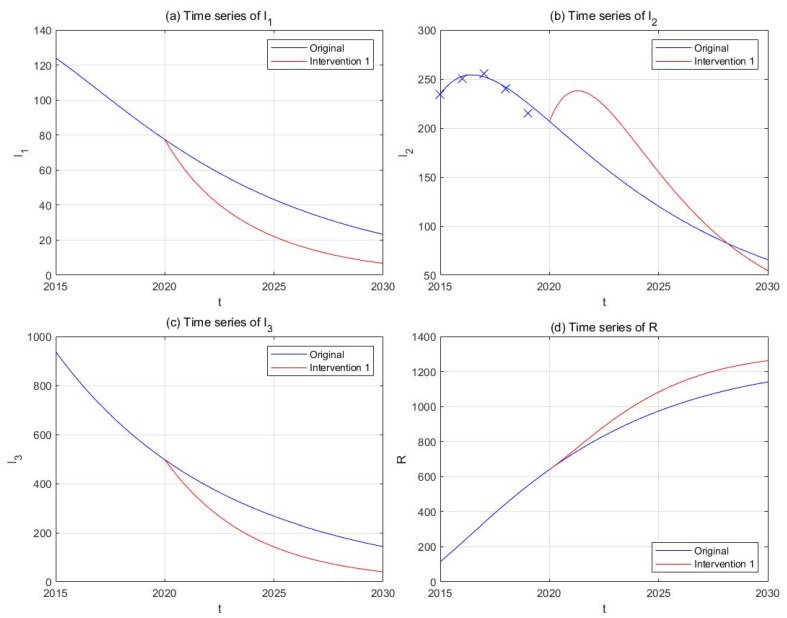
The effect of Intervention 3 (investigation and admission, red curves) in comparison with the original drug situation (blue curves). Subplots include time-series plots of light drug users (**a**), drug addicts undergoing treatment (**b**), hidden drug addicts (**c**), and recovered individuals (**d**).

In contrast to preventive strategies, we now consider improving the intensity of investigation of hidden drug users and their admission into drug rehabilitation facilities. We assume that the transfer rates $k_{1}$ and α are proportional to the intensity of investigation and admission, and that doubling the frequency and budgets of such activities, as well as the number of rehabilitation facilities, could increase $k_{1}$ and α by 100%. Supposing this change of parameters takes place since the end of 2020, we obtained the simulation results shown in Figure 10. It could be observed that the number of light drug users (−71.18%) and hidden drug addicts (−71.44%) undergoes sharp decreases compared to the original curves, and that of drug addicts in treatment $I_{2}$, though experiencing a temporary increase, ends up lower than the original curve (−16.82%).

(4) Intervention 4: moderate investigation

**Figure 11:**
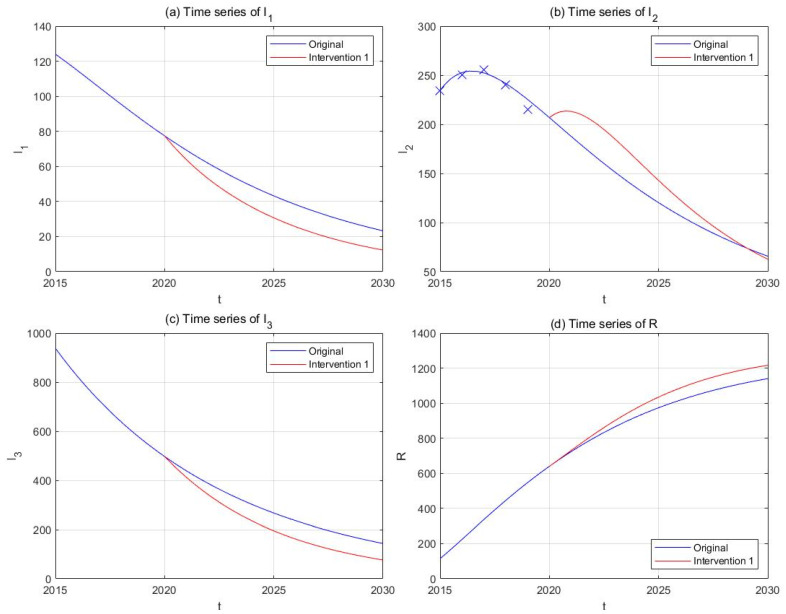
The effect of Intervention 4 (regional investigation, red curves) in comparison with the original drug situation (blue curves). Subplots include time-series plots of light drug users (**a**), drug addicts undergoing treatment (**b**), hidden drug addicts (**c**), and recovered individuals (**d**).

On account of the available police strengths and limited rehabilitation capacities of the detoxification facilities, we might consider an alternative approach instead of a thorough search of hidden drug users for the time being [67]. Hence, in this intervention strategy, the frequency and budgets of investigation activities, as well as the number of rehabilitation facilities are increased by 50%, corresponding to increases of 50% in $k_{1}$ and α. Repeat the rest of the procedures, and we obtain the results in Figure 11. A considerable drop is still observed in the $I_{3}$ compartment (−46.66%), and the final number of $I_{2}$ is also smaller than its original counterpart (−4.78%).

(5) Comparison of the interventions

In order to visually compare the effects of different intervention strategies on drug control, we tabulate the final numbers of all compartments in 2030 and their relative growth compared to the original curves in Table 4. The results show the high efficiency of Interventions 3 and 4 in reducing the number of drug users, especially hidden drug addicts. For instance, though possessing proximate basic reproduction numbers, Interventions 1 and 3 generated totally diverse outcomes in that the declining percentage of hidden drug addicts in Intervention 3 (−71.44%) was around 7 times larger than that in Intervention 1 (9.58%). Similar situations are also observed for Interventions 2 and 4.

## 6. Discussion

In this article, we propose a drug epidemic model with a complete addiction–rehabilitation–recovery process, which assumes the conversion of susceptible individuals into light drug users under the influence of drug addicts undergoing treatment and hidden drug addicts. Unlike many previous studies, we discard unrealistic assumptions such as self-detoxification without treatment or permanent immunity to drugs granted by “vaccines” or education [49,57]. We have acquired qualitative results of the dynamical behaviors of the model, including the feasible region, basic reproduction number, global asymptotic stabilities of the drug-free equilibrium and the drug-persistent equilibrium, as well as sensitivity analysis realized through normalized forward sensitivity indices. Subsequently, we applied the model to the drug epidemic in China and obtained the numerical simulation results via curve fitting and projections. The results show significant decreases in the numbers of all groups of drug users, including light drug users, drug addicts undergoing treatment, and hidden drug addicts. Should the model dynamics remain undisturbed, the predicted drug shrink in the following decade will be a positive signal to the accumulative anti-drug efforts by the Chinese government and public health workers, and it is in accordance with the 2030 Agenda for Sustainable Development by the United Nations [1].

One of the most interesting results could be observed in Section 5.4, where Interventions 3 and 4, which correspond to increasing transfer rates $k_{1}$ and α, turned out to be more efficient in reducing the numbers of existing drug users (including light drug users, drug addicts undergoing treatment, and hidden drug addicts) as well as the basic reproduction number than Interventions 1 and 2 (corresponding to lowering the effective contact rates $\beta_{1}$ and $\beta_{2}$). This conclusion is in accordance with the sensitivity analyses in Section 4, in that the parameters $\beta_{2}=3.86e-7,\alpha =0.124$ acquired through curve fitting correspond to the bottom-left corner of Figure 4b, where the basic reproduction number $R_{0}$ is more sensitive to changes in α than in $\beta_{2}$. Likewise, it can be shown that $R_{0}$ is far more sensitive to $\beta_{2}$ than to $k_{1}$ or $\beta_{1}$. As a consequence, it would not be surprising that a combination of α and $k_{1}$ (Interventions 3 and 4) is slightly more efficient in reducing $R_{0}$ than the combination of $\beta_{1}$ and $\beta_{2}$ (Interventions 1, 2). The conclusions above seem to contradict one of the most frequently stated conclusions that “prevention is better than cure” made by several previous studies [18,20,21] at first glimpse, but they are actually different aspects of the drug epidemic. Basic reproduction number $R_{0}$ focuses on the capability of new addicts to convert susceptible individuals into new initiates, whose effect on the existing drug addicts are realized through complex dynamical processes. On the contrary, transfer rates α and $k_{1}$ directly act on the existing number of hidden drug addicts and light drug users, and they proved to be efficient in controlling the scale of the drug epidemic. A direct proof of this phenomenon is the slight effect of Intervention 1 or 2 on the number of hidden drug addicts compared to that of Intervention 3 or 4, despite their similar basic reproduction numbers.

Another fact worth noticing is the tiny value of the basic reproduction number ($R_{0}=0.087256$ in the baseline). The calculation of this value was based on the formulation obtained in Section 3.2 and the parameter values acquired through curve fitting. Since we used the historical data of 2015−2018 to ascertain the parameter values and the data of 2019 to verify the projected trend, the result showed an overall well goodness of fit, and we have reasons to believe that this value of $R_{0}$ is an authentic manifestation of the drug situation in China for the modeling period. Given the illicit nature of drug-taking behaviors in China and the low reported number of existing drug users in official data, the scale of drug epidemic is by no means comparable to those of infectious diseases, and it seems reasonable that the $R_{0}$ of drug-using behaviors is smaller than those of infectious diseases by an order of magnitude [11]. Based on this $R_{0}$ and the already-decreasing number of drug users observed since 2018, it would not be difficult to understand the larger impact of Intervention 3 or 4 compared with Intervention 1 or 2 on the already-shrinking drug epidemic. In addition, it should also be noted that the number of drug addicts undergoing treatment in Interventions 3 and 4 even ended up lower than the original curves, and we owe this fact to the relatively higher value of $\beta_{2}$ compared with $\beta_{1}$, as well as the relevant dynamical processes. We believe the discussion above partially explains the observed results, and in the meantime, it offers new insights into formulations of anti-drug strategies and policies. The basic reproduction number $R_{0}$ is still an important threshold value for determination of the persistence of the drug epidemic, but cautions should be taken when choosing the appropriate strategy to further eliminate drug spread.

Our study possesses the following novelties: (1) a complete addiction–rehabilitation–recovery process, without unrealistic assumptions such as self-detoxification or permanent immunity; (2) application to the historical data of drug users in China and projection to the future; (3) novel insights in discussions of intervention strategies to accelerate the reduction of existing drug users. Similar to all existing modeling research studies, we acknowledge that our present study bears certain limitations. Above all, the assumption of permanent abstinence and absence of a relapse process is adopted for ease of analysis and application of the model, which is a major contradiction with the real situation around the world [76]. Secondly, the scarcity of historical data occurred due to former scopes of statistics adopted by the NNCC, which only provided accumulative numbers of drug users, and there was no other direct source available to secure the data of interest. Drug-use patterns and demographic characteristics may differ greatly among different subgroups divided according to age, gender, drug type consumed, etc., which requires advanced mathematical tools such as stratified models or partial differential equations [24,25,26,27,28,29,30,31,32,33]. Despite these limitations, our model still offers a universally applicable tool for prediction and analysis of the drug situation, and the complex issues listed above will be considered in our future research.

## 7. Conclusions

In this study, we have formulated a drug epidemic model with a complete addiction–rehabilitation–recovery process, which allows the generation of new initiates under the influence of drug addicts undergoing treatment and hidden drug addicts. We have established the basic properties of the model system, including the existence, uniqueness, and positivity of the solution, the forward-invariance of the feasible region, and the formulation of the basic reproduction number. We have shown that the drug-free equilibrium is globally asymptotically stable when $R_{0}\leq 1$, and the drug-persistent equilibrium is globally asymptotically stable when $R_{0}>1$. We have also carried out sensitivity analysis based on normalized forward sensitivity indices and numerical simulations, and we found that the relative efficiencies of parameters in adjusting $R_{0}$ can be debatable. Based on the established qualitative results, we use the model to simulate the drug epidemic in China, generate projections of the future, and provide in-depth discussions of intervention strategies. The simulation results show that the drug epidemic undergoes significant decreases in the following decade, and it would be more efficient to strengthen the investigation and admission of implicit drug users in order to pace up the elimination of drug spread. However, it should also be noticed that our model is a simplification of the real situation and can be further enhanced in its mathematical form. Further research could take into account the heterogeneous nature of the human society and incorporate complexities (e.g., delayed diffusive equations, multi-layer stochastic equations, and co-transmission models of various types of drugs) into their models. Moreover, regional-level modeling studies could also be carried out based on the availability of historical data, which arouses the needs for co-operation among epidemiologists, public health specialists, and governmental authorities.

## Figures and Tables

**Table 1:** Descriptions of model variables and parameters.

Variable/ Parameter	Description
S(t)	The susceptible individuals at time *t*
$I_{1}\left(t\right)$	Light drug users at time *t*
$I_{2}\left(t\right)$	Drug addicts undergoing treatment at time *t*
$I_{3}\left(t\right)$	Hidden drug addicts at time *t*
R(t)	Recovered individuals at time *t*
λ	Inflow rate into the susceptible individuals
μ	Natural death rate
$\mu_{d}$	Additional death rate resulting from drug abuse
$\beta_{1}$	Effective contact rate between drug addicts in treatment and susceptibles
$\beta_{2}$	Effective contact rate between hidden drug addicts and susceptibles
$k_{1}$	Progression rate from light drug users to drug addicts in treatment
$k_{2}$	Progression rate from light drug users to hidden drug addicts
α	Discovery and admission rate from hidden addicts to addicts in treatment
r	Recovery rate of drug addicts undergoing treatment

**Table 2:** Historical data of population aged 15−64, number of existing drug users, and former drug users who had remained abstinent for three or more years during 2015−2019.

Year	Population Aged 15−64 *	Existing Drug Users *	Former Drug Users Abstinent for ≥3 Years *
2015	100,361	234.5	114.8
2016	100,260	250.5	141.1
2017	99,829	255.3	167.9
2018	99,357	240.4	207.3
2019	98,914	214.8	253.3

* The unit of all numbers is 10,000 people.

**Table 3:** Parameter units, value ranges, final values used, and sources.

Parameter	Unit	Range	Value	Source
λ	10 thousand people/year	(235, 610)	400	Estimated from [71]
μ	/year	(0.0064, 0.00716)	0.007	[71]
$\mu_{d}$	/year	(0.021, 0.102)	0.025	[39,44]
$\beta_{1}$	/10 thousand people*year	(1 × 10^−9^, 1 × 10^−6^)	1.2481 × 10^−7^	Curve fit
$\beta_{2}$	/10 thousand people*year	(1 × 10^−9^, 1 × 10^−6^)	3.8611 × 10^−7^	Curve fit
$k_{1}$	/year	(0.05, 0.3)	0.176	Curve fit
$k_{2}$	/year	(0.05, 0.5)	0.2	[23,25,72]
α	/year	(0.05, 0.6)	0.124	Curve fit
r	/year	(0.33,0.6)	0.45	Estimated from [60]

**Table 4:** Comparison of Interventions 1−4 and their corresponding basic reproduction numbers.

	Original		Intervention 1		Intervention 2		Intervention 3		Intervention 4	
	2020 *	2030 *	2030 *	Increase (%)	2030 *	Increase (%)	2030 *	Increase (%)	2030 *	Increase (%)
S	97,368.48	94,533.21	94,592.85	0.06%	94,572.98	0.04%	94,566.47	0.04%	94,552.61	0.02%
$I_{1}$	77.42	23.28	11.81	−49.27%	15.55	−33.20%	6.71	−71.18%	12.31	−47.12%
$I_{2}$	206.86	65.70	57.30	−12.79%	60.10	−8.52%	54.65	−16.82%	62.56	−4.78%
$I_{3}$	496.84	143.57	129.81	−9.58%	134.40	−6.39%	41.00	−71.44%	76.58	−46.66%
R	639.97	1140.08	1116.05	−2.11%	1124.13	−1.40%	1261.71	10.67%	1216.28	6.68%
$R_{0}$	0.087256		0.043628		0.058462		0.042853		0.057216	

* The unit of all numbers of compartments is 10,000 people.

## Data Availability

Publicly available datasets were analyzed in this study. The data can be found in the following link: [http://www.nncc626.com/2020-06/24/c_1210675813.htm] and the extended links therein.

## Appendix A

**Proof of Theorem 1.** To investigate the local asymptotic stability of the drug-free equilibrium $E_{0}=\left(\frac{\lambda }{\mu },0,0,0,0\right)$, we obtain the Jacobian matrix associated with System (1) evaluated at $E_{0}$:

$$J|{}_{E_{0}}=\left[\begin{array}{ccccc} -\mu & 0 & -\frac{\beta_{1}\lambda }{\mu } & -\frac{\beta_{2}\lambda }{\mu } & 0\\ 0 & -\left(k_{1}+k_{2}+\mu \right) & \frac{\beta_{1}\lambda }{\mu } & \frac{\beta_{2}\lambda }{\mu } & 0\\ 0 & k_{1} & -\left(r+\mu \right) & \alpha & 0\\ 0 & k_{2} & 0 & -\left(\alpha +\mu +\mu_{d}\right) & 0\\ 0 & 0 & r & 0 & -\mu \end{array}\right].$$

Through direct calculations, we see that the eigenvalues of $J|{}_{E_{0}}$ are −μ (multiplicity 2) and the solution to the cubic equation $a_{3}x^{3}+a_{2}x^{2}+a_{1}x+a_{0}=0$, where

$$a_{3}=1,a_{2}=\alpha +3\mu +\mu_{d}+k_{1}+k_{2}+r,\;a_{1}=\left(k_{1}+k_{2}+\mu \right)\left(\alpha +\mu +\mu_{d}\right)+\left(r+\mu \right)\left(k_{1}+k_{2}+2\mu +\alpha +\mu_{d}\right)-\left(k_{1}\beta_{1}+k_{2}\beta_{2}\right)\frac{\lambda }{\mu },\;a_{0}=-\frac{\beta_{1}\lambda }{\mu }\left[k_{2}\alpha +k_{1}\left(\alpha +\mu +\mu_{d}\right)\right]+\left(r+\mu \right)\left[\left(k_{1}+k_{2}+\mu \right)\left(\alpha +\mu +\mu_{d}\right)-k_{2}\beta_{2}\frac{\lambda }{\mu }\right].$$

It is obvious that $a_{3}>0,a_{2}>0$, and from

$$R_{0}=\frac{\left[\left(\alpha +\mu +\mu_{d}\right)k_{1}+\alpha k_{2}\right]\beta_{1}+k_{2}\left(r+\mu \right)\beta_{2}}{\left(k_{1}+k_{2}+\mu \right)\left(r+\mu \right)\left(\alpha +\mu +\mu_{d}\right)}\cdot \frac{\lambda }{\mu }<1,$$

we can easily obtain that $a_{0}>0$. It can be deduced that

$$\frac{k_{1}\beta_{1}\lambda }{\mu }<\left(k_{1}+k_{2}+\mu \right)\left(r+\mu \right)\;\mathrm{and}\;\frac{k_{2}\beta_{2}\lambda }{\mu }<\left(k_{1}+k_{2}+\mu \right)\left(\alpha +\mu +\mu_{d}\right).$$

Hence, we have $a_{1}>\left(r+\mu \right)\left(\alpha +\mu +\mu_{d}\right)>0$. Moreover, it can also be obtained that

$$a_{2}a_{1}-a_{3}a_{0}>\left(\alpha +\mu +\mu_{d}\right)\left(r+\mu \right)\left(2k_{1}+2k_{2}+4\mu +r+\alpha +\mu_{d}\right)+k_{2}\alpha \beta_{1}\frac{\lambda }{\mu }>0.$$

According to Routh–Hurwitz criterion, all eigenvalues of $J|{}_{E_{0}}$ have negative real parts when $a_{0},a_{1},a_{2},a_{3}>0$ and $a_{2}a_{1}-a_{3}a_{0}>0$, which leads to the conclusion that $E_{0}$ is locally asymptotically stable when $R_{0}<1$ [64]. To prove the global asymptotic stability of $E_{0}$, we need to construct a suitable Lyapunov function. By applying the method introduced by Li et al. [77], we define b≥0 as the left eigenvector of the non-negative matrix $V^{-1}F$ with respect to the eigenvalue $R_{0}$. We can easily obtain that $b=\left(0,\beta_{1},\beta_{2}\right)$ is a suitable eigenvector and further establish the following Lyapunov function:

(A1) $$L=bV^{-1}x=\frac{\mu R_{0}}{\lambda }I_{1}+\frac{\beta_{1}}{r+\mu }I_{2}+\frac{\alpha \beta_{1}+\left(r+\mu \right)\beta_{2}}{\left(r+\mu \right)\left(\alpha +\mu +\mu_{d}\right)}I_{3}.$$

Compute the time-derivative of L along the trajectory of System (1):

$$\frac{dL}{dt}=\frac{\mu R_{0}}{\lambda }\cdot \frac{dI_{1}}{dt}+\frac{\beta_{1}}{r+\mu }\cdot \frac{dI_{2}}{dt}+\frac{\alpha \beta_{1}+\left(r+\mu \right)\beta_{2}}{\left(r+\mu \right)\left(\alpha +\mu +\mu_{d}\right)}\cdot \frac{dI_{3}}{dt}\;=\frac{\mu R_{0}}{\lambda }\left[\beta_{1}SI_{2}+\beta_{2}SI_{3}-\left(k_{1}+k_{2}+\mu \right)I_{1}\right]+\frac{\beta_{1}}{r+\mu }\left[k_{1}I_{1}+\alpha I_{3}-\left(r+\mu \right)I_{2}\right]\;+\frac{\alpha \beta_{1}+\left(r+\mu \right)\beta_{2}}{\left(r+\mu \right)\left(\alpha +\mu +\mu_{d}\right)}\left[k_{2}I_{1}-\left(\alpha +\mu +\mu_{d}\right)I_{3}\right].$$

Through tedious algebraic manipulations, we acquire the coefficients of $I_{1}∼I_{3}$. Coefficient of $I_{1}$:

$$\frac{\beta_{1}k_{1}}{r+\mu }+\frac{\alpha k_{2}\beta_{1}+\left(r+\mu \right)k_{2}\beta_{2}}{\left(r+\mu \right)\left(\alpha +\mu +\mu_{d}\right)}-\left(k_{1}+k_{2}+\mu \right)\frac{\mu R_{0}}{\lambda }=0,$$

Coefficient of $I_{2}$:

$$\frac{\mu R_{0}}{\lambda }\beta_{1}S-\beta_{1}\leq \beta_{1}\left(R_{0}-1\right)\leq 0$$

when $R_{0}\leq 1$, Coefficient of $I_{3}$:

$$\frac{\mu R_{0}}{\lambda }\beta_{2}S-\beta_{2}\leq \beta_{2}\left(R_{0}-1\right)\leq 0$$

when $R_{0}\leq 1$. The equalities above can be satisfied only when $R_{0}=1$, which corresponds to the drug-free equilibrium $E_{0}=\left(\frac{\lambda }{\mu },0,0,0,0\right)$. By LaSalle’s invariance principle, the largest invariant set in

$$\Gamma =\left\{\left(S,I_{1},I_{2},I_{3},R\right)\in {\mathbb{R}}_{+}^{5};0\leq S+I_{1}+I_{2}+I_{3}+R\leq \frac{\lambda }{\mu }\right\}$$

is reduced to the singleton $E_{0}$ in this case. Hence, the unique drug-free equilibrium is globally asymptotically stable; thus, it completes the proof. □

## Appendix B

**Proof of Theorem 2.** The Jacobian matrix method seems impractical for the analysis of local stability of the drug-persistent equilibrium $E^{*}$, since determination of the eigenvalues requires solving a quartic equation, which is difficult to handle even for Routh–Hurwitz criterion. Alternatively, we choose the bifurcation method based on center manifold theory by Castillo-Chavez and Song [78]. Consider System (1) as an ODE system with bifurcation parameter φ:

$$\frac{dx}{dt}=f\left(x,\phi \right),f:{\mathbb{R}}^{5}\times \mathbb{R}\to {\mathbb{R}}^{5},f\in {\mathbb{C}}^{2}\left({\mathbb{R}}^{5}\times \mathbb{R}\right).$$

The choice of the bifurcation parameter φ should satisfy $f\left(E_{0},\phi \right)\equiv 0$ for ∀ϕ. In this case, $\beta_{1}$ is obviously an eligible parameter for ϕ. To proceed, we substitute the variables as $S=x_{1},I_{1}=x_{2},I_{2}=x_{3},I_{3}=x_{4},R=x_{5}$, and System (1) changes to:

(A2) $$f_{1}=\lambda -\beta_{1}x_{1}x_{3}-\beta_{2}x_{1}x_{4}-\mu x_{1}\;f_{2}=\beta_{1}x_{1}x_{3}+\beta_{2}x_{1}x_{4}-\left(k_{1}+k_{2}+\mu \right)x_{2}\;f_{3}=k_{1}x_{2}+\alpha x_{4}-\left(r+\mu \right)x_{3}\;f_{4}=k_{2}x_{2}-\left(\alpha +\mu +\mu_{d}\right)x_{4}\;f_{5}=rx_{3}-\mu x_{5}.$$

Set $R_{0}=\frac{\left[\left(\alpha +\mu +\mu_{d}\right)k_{1}+\alpha k_{2}\right]\beta_{1}+k_{2}\left(r+\mu \right)\beta_{2}}{\left(k_{1}+k_{2}+\mu \right)\left(r+\mu \right)\left(\alpha +\mu +\mu_{d}\right)}\cdot \frac{\lambda }{\mu }=1$, and based on previous results of the Jacobian matrix $J|{}_{E_{0}}$, it is not difficult to find that zero is an eigenvalue of $J|{}_{E_{0}}$, and all other eigenvalues have negative real parts. Hence, the criterion for the application of the bifurcation method is met. Subsequently, we compute the right and left eigenvectors corresponding to zero eigenvalue. The right eigenvector is given by $\vec{w}={\left(w_{1},w_{2},w_{3},w_{4},w_{5}\right)}^{T}$, where

$$w_{1}=-\frac{k_{1}+k_{2}+\mu }{\mu }\cdot w_{2},w_{3}=\frac{\mu \left(k_{1}+k_{2}+\mu \right)\left(\alpha +\mu +\mu_{d}\right)-k_{2}\beta_{2}\lambda }{\left(\alpha +\mu +\mu_{d}\right)\beta_{1}\lambda }\cdot w_{2},\;w_{4}=-\frac{k_{2}}{\alpha +\mu +\mu_{d}}\cdot w_{2},w_{5}=\frac{\mu \left(k_{1}+k_{2}+\mu \right)\left(\alpha +\mu +\mu_{d}\right)-k_{2}\beta_{2}\lambda }{\left(\alpha +\mu +\mu_{d}\right)\beta_{1}\lambda \mu }\cdot rw_{2}.$$

The left eigenvalue is given by $\vec{v}=\left(0,v_{2},v_{3},v_{4},0\right)$, where

$$v_{3}=\frac{\beta_{1}\lambda }{\left(r+\mu \right)\mu }\cdot v_{2},v_{4}=\frac{\mu \left(k_{1}+k_{2}+\mu \right)\left(r+\mu \right)-k_{1}\beta_{1}\lambda }{\mu k_{2}\left(r+\mu \right)}\cdot v_{2}$$

Let $f_{k}$ be the kth equation of System (5), and according to the bifurcation method, $a=\sum_{k,i,j=1}^{5}v_{k}w_{i}w_{j}\frac{\partial^{2}f_{k}}{\partial x_{i}\partial x_{j}}\left(0,0\right)$, and $b=\sum_{k,i=1}^{5}v_{k}w_{i}\frac{\partial^{2}f_{k}}{\partial x_{i}\partial \phi }\left(0,0\right)$. The associated non-zero second-order partial derivatives around $\left(E_{0},0\right)$ are: $\frac{\partial^{2}f_{2}}{\partial x_{1}\partial x_{3}}=\frac{\partial^{2}f_{2}}{\partial x_{3}\partial x_{1}}=\beta_{1}$, $\frac{\partial^{2}f_{2}}{\partial x_{1}\partial x_{4}}=\frac{\partial^{2}f_{2}}{\partial x_{4}\partial x_{1}}=\beta_{2}$, $\frac{\partial^{2}f_{2}}{\partial x_{3}\partial \phi }=\frac{\lambda }{\mu }$. The rest of the second-order partial derivatives were all calculated to be 0 and are hence omitted. Take $v_{2}=w_{2}=1$, and it is easy to obtain that:

$$a=v_{2}\sum_{i,j=1}^{5}w_{i}w_{j}\frac{\partial^{2}f_{2}}{\partial x_{i}\partial x_{j}}\left(0,0\right)=2\left(w_{1}w_{3}\phi +w_{1}w_{4}\beta_{2}\right).$$

It is obvious that $w_{1}<0,w_{4}>0$, and given the condition of $R_{0}=1$, it is not hard to prove $w_{3}>0$. Hence, we have a<0 proved. On the other hand, we have:

$$b=v_{2}\sum_{i}^{5}w_{i}\frac{\partial^{2}f_{2}}{\partial x_{i}\partial \phi }\left(0,0\right)=\frac{w_{3}\lambda }{\mu }>0.$$

In conclusion, since a<0 and b>0, according to the theory of backward bifurcation, the model system experiences forward bifurcation, and the drug-persistent equilibrium is locally asymptotically stable for $R_{0}>1$ but close to one [40]. To prove the global stability of the drug-persistent equilibrium $E^{*}$, we now construct a Volterra-type Lyapunov function following the method described in [77]:

$$V=\frac{\mu R_{0}\left(r+\mu \right)}{\lambda }V_{1}+\beta_{1}V_{2}+\frac{\beta_{2}\left(r+\mu \right)+\alpha \beta_{1}}{\alpha +\mu +\mu_{d}}V_{3},$$

among which:

$$V_{1}=S-S^{*}-S^{*}\ln\frac{S}{S^{*}}+I_{1}-I_{1}^{*}-I_{1}^{*}\ln\frac{I_{1}}{I_{1}^{*}}\;V_{2}=I_{2}-I_{2}^{*}-I_{2}^{*}\ln\frac{I_{2}}{I_{2}^{*}},\;V_{3}=I_{3}-I_{3}^{*}-I_{3}^{*}\ln\frac{I_{3}}{I_{3}^{*}}.$$

The time-derivative of $V_{1}$ is given by:

$$\frac{dV_{1}}{dt}=\left(1-\frac{S^{*}}{S}\right)\frac{dS}{dt}+\left(1-\frac{I_{1}^{*}}{I_{1}}\right)\frac{dI_{1}}{dt}\;=\left(1-\frac{S^{*}}{S}\right)\left(\lambda -\beta_{1}SI_{2}-\beta_{2}SI_{3}-\mu S\right)+\left(1-\frac{I_{1}^{*}}{I_{1}}\right)\left[\beta_{1}SI_{2}+\beta_{2}SI_{3}-\left(k_{1}+k_{2}+\mu \right)I_{1}\right].$$

Based on System (3) at the drug-persistent equilibrium $E^{*}$, we obtain $\lambda =\beta_{1}S^{*}I_{2}^{*}+\beta_{2}S^{*}I_{3}^{*}+\mu S^{*}$ and $k_{1}+k_{2}+\mu =\frac{\beta_{1}S^{*}I_{2}^{*}+\beta_{2}S^{*}I_{3}^{*}}{I_{1}^{*}}$, and the equation above turns into:

$$\frac{dV_{1}}{dt}=\left(1-\frac{S^{*}}{S}\right)\left[\beta_{1}S^{*}I_{2}^{*}\left(1-\frac{SI_{2}}{S^{*}I_{2}^{*}}\right)+\beta_{2}S^{*}I_{3}^{*}\left(1-\frac{SI_{3}}{S^{*}I_{3}^{*}}\right)+\mu \left(S^{*}-S\right)\right]\;+\left(1-\frac{I_{1}^{*}}{I_{1}}\right)\left[\beta_{1}S^{*}I_{2}^{*}\left(\frac{SI_{2}}{S^{*}I_{2}^{*}}-\frac{I_{1}}{I_{1}^{*}}\right)+\beta_{2}S^{*}I_{3}^{*}\left(\frac{SI_{3}}{S^{*}I_{3}^{*}}-\frac{I_{1}}{I_{1}^{*}}\right)\right]\;∵\Theta_{1}=\left(1-\frac{S^{*}}{S}\right)\left(S^{*}-S\right)\leq 0\;∴\frac{dV_{1}}{dt}\leq \beta_{1}S^{*}I_{2}^{*}\left(2-\frac{S^{*}}{S}+\frac{I_{2}}{I_{2}^{*}}-\frac{I_{1}}{I_{1}^{*}}-\frac{I_{1}^{*}SI_{2}}{I_{1}S^{*}I_{2}^{*}}\right)+\beta_{2}S^{*}I_{3}^{*}\left(2-\frac{S^{*}}{S}+\frac{I_{3}}{I_{3}^{*}}-\frac{I_{1}}{I_{1}^{*}}-\frac{I_{1}^{*}SI_{3}}{I_{1}S^{*}I_{3}^{*}}\right).$$

Let $F=2-\frac{S^{*}}{S}+\frac{I_{2}}{I_{2}^{*}}-\frac{I_{1}}{I_{1}^{*}}-\frac{I_{1}^{*}SI_{2}}{I_{1}S^{*}I_{2}^{*}}$, $G=2-\frac{S^{*}}{S}+\frac{I_{3}}{I_{3}^{*}}-\frac{I_{1}}{I_{1}^{*}}-\frac{I_{1}^{*}SI_{3}}{I_{1}S^{*}I_{3}^{*}}$, and define $D\left(x\right)=-\frac{x}{x^{*}}+\ln\frac{x}{x^{*}}$, Φ(a)=1−a+lna as in [78]. It is easy to prove Φ(a)=1−a+lna≤0 for ∀a>0, and the equality can be reached only when a=1. Thus, the expressions above can be rewritten as:

$$F=\Phi \left(\frac{S^{*}}{S}\right)-\ln\left(\frac{S^{*}}{S}\right)+\Phi \left(\frac{I_{1}^{*}SI_{2}}{I_{1}S^{*}I_{2}^{*}}\right)-\ln\left(\frac{I_{1}^{*}SI_{2}}{I_{1}S^{*}I_{2}^{*}}\right)+\frac{I_{2}}{I_{2}^{*}}-\frac{I_{1}}{I_{1}^{*}}\;\leq \frac{I_{2}}{I_{2}^{*}}-\frac{I_{1}}{I_{1}^{*}}-\ln\left(\frac{I_{1}^{*}I_{2}}{I_{1}I_{2}^{*}}\right)=D\left(I_{1}\right)-D\left(I_{2}\right),\;G=\Phi \left(\frac{S^{*}}{S}\right)-\ln\left(\frac{S^{*}}{S}\right)+\Phi \left(\frac{I_{1}^{*}SI_{3}}{I_{1}S^{*}I_{3}^{*}}\right)-\ln\left(\frac{I_{1}^{*}SI_{3}}{I_{1}S^{*}I_{3}^{*}}\right)+\frac{I_{3}}{I_{3}^{*}}-\frac{I_{1}}{I_{1}^{*}}\;\leq \frac{I_{3}}{I_{3}^{*}}-\frac{I_{1}}{I_{1}^{*}}-\ln\left(\frac{I_{1}^{*}I_{3}}{I_{1}I_{3}^{*}}\right)=D\left(I_{1}\right)-D\left(I_{3}\right).$$

Hence, it can be deduced that:

$$\frac{dV_{1}}{dt}\leq \beta_{1}S^{*}I_{2}^{*}\left[D\left(I_{1}\right)-D\left(I_{2}\right)\right]+\beta_{2}S^{*}I_{3}^{*}\left[D\left(I_{1}\right)-D\left(I_{3}\right)\right].$$

Similarly, the time-derivative of $V_{2}$ is given by:

$$\frac{dV_{2}}{dt}=\left(1-\frac{I_{2}^{*}}{I_{2}}\right)\frac{dI_{2}}{dt}=\left(1-\frac{I_{2}^{*}}{I_{2}}\right)\left[k_{1}I_{1}+\alpha I_{3}-\left(r+\mu \right)I_{2}\right].$$

Based on System (3) at the drug-persistent equilibrium $E^{*}$, we obtain $r+\mu =\frac{k_{1}I_{1}^{*}+\alpha I_{3}^{*}}{I_{2}^{*}}$, and the equation above turns into:

$$\frac{dV_{2}}{dt}=\left(1-\frac{I_{2}^{*}}{I_{2}}\right)\left[k_{1}I_{1}^{*}\left(\frac{I_{1}}{I_{1}^{*}}-\frac{I_{2}}{I_{2}^{*}}\right)+\alpha I_{3}^{*}\left(\frac{I_{3}}{I_{3}^{*}}-\frac{I_{2}}{I_{2}^{*}}\right)\right]\;=k_{1}I_{1}^{*}\left(\frac{I_{1}}{I_{1}^{*}}-\frac{I_{2}}{I_{2}^{*}}-\frac{I_{1}I_{2}^{*}}{I_{1}^{*}I_{2}}+1\right)+\alpha I_{3}^{*}\left(\frac{I_{3}}{I_{3}^{*}}-\frac{I_{2}}{I_{2}^{*}}-\frac{I_{2}^{*}I_{3}}{I_{2}I_{3}^{*}}+1\right)\;=k_{1}I_{1}^{*}\left[\frac{I_{1}}{I_{1}^{*}}-\frac{I_{2}}{I_{2}^{*}}+\Phi \left(\frac{I_{1}I_{2}^{*}}{I_{1}^{*}I_{2}}\right)-\ln\left(\frac{I_{1}I_{2}^{*}}{I_{1}^{*}I_{2}}\right)\right]+\alpha I_{3}^{*}\left[\frac{I_{3}}{I_{3}^{*}}-\frac{I_{2}}{I_{2}^{*}}+\Phi \left(\frac{I_{2}^{*}I_{3}}{I_{2}I_{3}^{*}}\right)-\ln\left(\frac{I_{2}^{*}I_{3}}{I_{2}I_{3}^{*}}\right)\right]\;\leq k_{1}I_{1}^{*}\left(\frac{I_{1}}{I_{1}^{*}}-\frac{I_{2}}{I_{2}^{*}}-\ln\frac{I_{1}}{I_{1}^{*}}+\ln\frac{I_{2}}{I_{2}^{*}}\right)+\alpha I_{3}^{*}\left(\frac{I_{3}}{I_{3}^{*}}-\frac{I_{2}}{I_{2}^{*}}-\ln\frac{I_{3}}{I_{3}^{*}}+\ln\frac{I_{2}}{I_{2}^{*}}\right).$$

Hence, it is easily obtained that $\frac{dV_{2}}{dt}\leq k_{1}I_{1}^{*}\left[D\left(I_{2}\right)-D\left(I_{1}\right)\right]+\alpha I_{3}^{*}\left[D\left(I_{2}\right)-D\left(I_{3}\right)\right]$. By similar algebraic manipulations, we can also obtain that:

$$\frac{dV_{3}}{dt}\leq k_{2}I_{1}^{*}\left(\frac{I_{1}}{I_{1}^{*}}-\frac{I_{3}}{I_{3}^{*}}-\ln\frac{I_{1}}{I_{1}^{*}}+\ln\frac{I_{3}}{I_{3}^{*}}\right)=k_{2}I_{1}^{*}\left[D\left(I_{3}\right)-D\left(I_{1}\right)\right].$$

As a consequence, the time-derivative of the Lyapunov function becomes:

$$\frac{dV}{dt}=\frac{\mu R_{0}\left(r+\mu \right)}{\lambda }\cdot \frac{dV_{1}}{dt}+\beta_{1}\cdot \frac{dV_{2}}{dt}+\frac{\beta_{2}\left(r+\mu \right)+\alpha \beta_{1}}{\alpha +\mu +\mu_{d}}\cdot \frac{dV_{3}}{dt},\;\leq \frac{\mu R_{0}\left(r+\mu \right)}{\lambda }\left\{\beta_{1}S^{*}I_{2}^{*}\left[D\left(I_{1}\right)-D\left(I_{2}\right)\right]+\beta_{2}S^{*}I_{3}^{*}\left[D\left(I_{1}\right)-D\left(I_{3}\right)\right]\right\}\;+\beta_{1}\left\{k_{1}I_{1}^{*}\left[D\left(I_{2}\right)-D\left(I_{1}\right)\right]+\alpha I_{3}^{*}\left[D\left(I_{2}\right)-D\left(I_{3}\right)\right]\right\}+\frac{k_{2}I_{1}^{*}\left[\beta_{2}\left(r+\mu \right)+\alpha \beta_{1}\right]}{\alpha +\mu +\mu_{d}}\left[D\left(I_{3}\right)-D\left(I_{1}\right)\right].\;\equiv 0$$

Hence, the right-hand side of the inequality is identically vanishing, and $\frac{dV}{dt}\leq 0$ holds whenever $R_{0}>1$. The equalities above can be satisfied only when $S=S^{*}$, $I_{1}=I_{1}^{*}$, $I_{2}=I_{2}^{*}$ and $I_{3}=I_{3}^{*}$, which corresponds to the drug-persistent equilibrium $E^{*}=\left(S^{*},I_{1}^{*},I_{2}^{*},I_{3}^{*},R^{*}\right)$. By LaSalle’s invariance principle, the largest invariant set in $\Gamma =\left\{\left(S,I_{1},I_{2},I_{3},R\right)\in {\mathbb{R}}_{+}^{5};0\leq S+I_{1}+I_{2}+I_{3}+R\leq \frac{\lambda }{\mu }\right\}$ is reduced to the singleton $E^{*}$ in this case. Hence, the drug-persistent equilibrium is globally asymptotically stable, thus completing the proof. □
